# Brassinosteroids and Response of Plants to Heavy Metals Action

**DOI:** 10.3389/fpls.2016.00629

**Published:** 2016-05-09

**Authors:** Iwona Rajewska, Marta Talarek, Andrzej Bajguz

**Affiliations:** Faculty of Biology and Chemistry, Institute of Biology, University of Białystok, BialystokPoland

**Keywords:** antioxidants, brassinosteroids, heavy metals, phytochelatins, phytohormones

## Abstract

Brassinosteroids (BRs) are a widespread group of plant hormones. These phytohormones play a crucial role in the regulation of growth and development of various plant species, and they demonstrate high biological activity. BRs are considered to demonstrate protective activity in the plants exposed to various stresses. Due to rapid industrialization and urbanization, heavy metals have become one of the most important plant stressors. In plants, accumulation of heavy metals beyond the critical levels leads to oxidative stress. However, BRs may inhibit the degradation of lipids, resulted from the overproduction of reactive oxygen species under stress conditions, and increase the activity of antioxidants. They also have the ability to promote phytochelatins synthesis.

## Introduction

Brassinosteroids (BRs) are plant hormones that belong to the class of polyhydroxy steroids. BRs can regulate multiple physiological functions, such as embryogenesis and seed germination, cell divisions and elongation, development of thecae, microspore germination and growth of pollen tubes, differentiation of tracheal elements, polarization of cellular membranes as well as leaf senescence and dying (Clouse and Sasse, 1998). For the past one decade, there has been a significant progress in our understandings’ of BRs biosynthesis, degradation, and their signaling, as well as their versatile role in growth and developmental processes in plants (Bajguz, 2007, 2012; Zhao and Li, 2012; Wang et al., 2014; Zhabinskii et al., 2015).

This paper highlights the progress in the field of BRs research with more emphasis on new key players identified in the growing implications of BRs research in the tolerance of metal stress.

## Brassinosteroids VS. Heavy Metal Stress

Industrial revolution and the activity of man lead to more and more serious pollution of the environment with metals. They finally get infiltrated in the food chain, which results into the degradation of ecosystem. Plants have the amazing ability to take up and accumulate metals from the environment. Although some metals are essential for most redox reactions fundamental for cellular functions, still, high concentrations of all metals, even those essential for plant growth and metabolism, cause toxic effects. The impact of toxic metals on plants mostly involves interaction with functional groups of molecules in cells, particularly proteins, and polynucleotides (Chary et al., 2008). These effects may include growth inhibition, the reduction in net photosynthetic rate, lowering the contents of photosynthetic pigments, carbohydrates and proline, increased content of malondialdehyde. During oxidative stress, several plant hormones play a key role. BRs, however, not only regulate different physiological and morphogenetic responses in plants but also help in reducing various biotic and abiotic stresses (Krishna, 2003; Bajguz and Hayat, 2009; Vardhini, 2016).

## BRs Response to Plant Development

Accumulation of metals such as cadmium, copper, lead, and zinc after the application of BRs has been investigated in different cultivated plants, e.g., tomato, barley, and radish (Anuradha and Rao, 2007a,b; Hayat et al., 2010; Hasan et al., 2011; Ramakrishna and Rao, 2013, 2015). It has been also proved that after the application of 24-epibrassinolide (EBL, one of the BRs) the lead content in a beet root is even 50% lower than in plants treated with metal alone, since this hormone considerably reduces the absorption of this metal (Khripach et al., 1999). When using BRs for Indian mustard (*Brassica juncea*) seeds before the germination and then exposing them against copper stress, reduced uptake and accumulation of copper was observed, as well as improvement in shoot generation and biomass production also occurred (Sharma et al., 2007). Changes in the content of ions/metals under the influence of BRs depend on the stage of development during which they were applied to the plant. Research conducted so far shows that cultures of *Chlorella vulgaris* treated with metals and BRs display lower bioaccumulation of metals than those which were only exposed to metals. After reducing the accumulation of metals, BRs stimulate the growth, and development of *C. vulgaris*. The culture of *C. vulgaris* exposed to metals shows that BRs prevent the loss of chlorophyll, sugar, and protein and improve the syntheses of phytochelatins (PC; Bajguz, 2000, 2002). Moreover, it has been proved that brassinolide promotes the growth of mung bean seedlings under aluminum stress (Abdullahi et al., 2003). In addition, EBL considerably increases the fresh mass of shoots and roots as well as chlorophyll content in mung beans under aluminum stress (Ali et al., 2008a). We also know that the use of 28-homobrassinolide (HBL) in Indian mustard exposed to nickel activity improves seed germination as well as the lengths of shoots and roots, both under Ni stress and otherwise (Yusuf et al., 2011). BR eliminates the toxic effect of cadmium on photochemical pathways in rape cotyledons, mostly by means of alleviating the damage to reaction centers and O_2_ evolving complexes and by ensuring efficient electron transport (Janeczko et al., 2005).

Recent studies show that HBL can reduce metal stress in plants, such as Indian mustard, radish, wheat, and maize (Anuradha and Rao, 2007b; Bhardwaj et al., 2007; Hayat et al., 2007; Sharma et al., 2010). It is also known that HBL regulates the activities of various enzymes involved in photosynthesis and plant defense mechanisms in wheat and Indian mustard exposed to different abiotic stresses (Hayat et al., 2007). It has been revealed that pre-soaked seeds in HBL improves seedling growth as well as chlorophyll *a* content under the exposure of metal. Moreover, it has been shown that the increased uptake of Cr^2+^ in radish or rice seedlings lowers considerably after treatment with BR, thus reducing chromium toxicity (Sharma et al., 2011, 2016).

## Reactive Oxygen Species and Antioxidants

**FIGURE 1:**
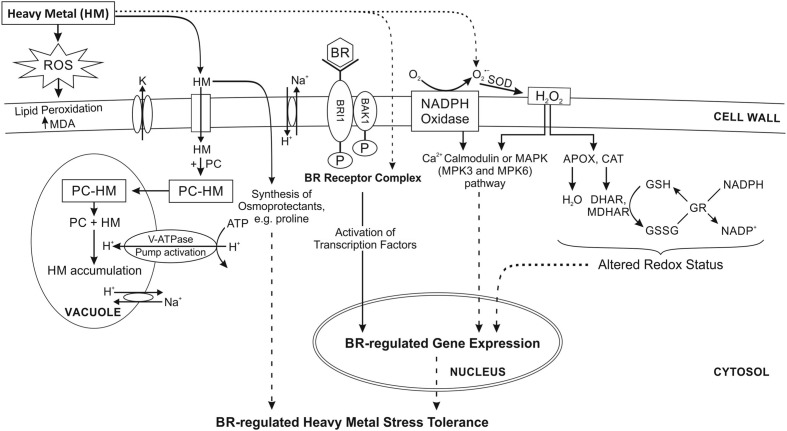
**Proposed mechanism of BRs regulation of heavy metal stress tolerance in plants.** Exogenously applied BRs enhance the tolerance to oxidative, and heavy metals stress. It is accompanied by the accumulation of H_2_O_2_ and the enhancement of antioxidants enzymes, which scavenge excessive ROS. It remains largely unknown how BRs induce ROS production and upregulates antioxidant defense. MAPKs are triggered as a result of ROS accumulation. However, there is no evidence on direct association or interaction with BRs. The molecular mechanism of BR-induced plant stress tolerance remains poorly understood. BRs mitigate the negative impact of metal toxicity by enhancing lipid peroxidation, sodium and potassium ion concentrations, proline content, total osmolyte content, and level of antioxidants. The chelation of the metal ion by PC is other mechanisms for metal detoxification. APOX, ascorbate peroxidise; BAK1, BRI1-associated kinase1; BR, brasinosteroid; BRI1, brassinosteroid insensitive1; CAT, catalase; DHAR, dehydroascorbate reductase; GR, glutathione reductase; GSH, glutathione; GSSG, glutathione disulphide; HM, heavy metal; K, potassium; MAPK, mitogen-activated protein kinases; MDA, malondialdehyde; MDHAR, monodehydroascorbate reductase; P, phosphate; PC, phytochelatins; ROS, reactive oxygen species; V-ATPase, vacuolar H^+^-ATPase. Dotted line shows the possible mechanism, which remains to be explored. Modified from Sharma et al. (2011).

Quick development of industry and the resultant more and more serious pollution of the environment with metals has been the reason for extensive investigation of potential mechanisms of adaptation to this stressor. Negative effects of metals on plants are first of all manifested in growth inhibition. The basic toxic activity of metal ions results from oxidative stress, related with generating reactive oxygen species (ROS; Krishna, 2003). In response to metal stress, calcium-dependent protein kinases, calmodulins, calmodulin-like proteins, and calcineurin B-like proteins are also activated as important Ca^2+^ sensors in plants (Ludwig et al., 2004). Experiments carried out with the use of *Arabidopsis* have shown that in response to Cd, two mitogen-activated protein kinases (MAPKs), MPK3 and MPK6 are triggered as a result of ROS accumulation (Liu et al., 2010) (**Figure 1**). However, to this date there is no evidence on direct association or interaction with BRs. Toxicity related with metal activity leads to over-production of ROS and thus causes oxidative damage resulting in membrane destruction, which in turn affects the levels of antioxidants and metallozymes or antioxidant enzymes (Pandey et al., 2005). Experiments conducted by Ali et al. (2008b) using mustard plants show that the influence of BR on the antioxidative system is more evident under stress conditions, suggesting that an elevated level of antioxidative system partially increases the plants’ tolerance to salt and/or nickel stress and thus protects the photosynthesis and plant growth. The activities of catalase (CAT), ascorbate peroxidise (APOX), and superoxide dismutase (SOD), as well as the content of proline, increased in cadmium-treated plants, especially in mustard plants simultaneously supplemented with HBL. Similar results were obtained for mung bean plants exposed to aluminum with the simultaneous spraying of EBL or HBL. Spraying the leaves with BRs significantly enhanced the contents of antioxidant enzymes and proline in aluminum-stressed mung bean seedlings (Ali et al., 2008a). Other reports have also showed that BR application can modify the activity of antioxidant enzymes in maize, mustard, radish, wheat, and rice exposed to metal stress (Sharma et al., 2007, 2010, 2016). According to the AsA-GSH Cycle/Asada Halliwell Pathway ascorbate is essential for ROS scavenging. Its cellular pool is maintained stable by dehydroascorbate reductase (DHAR) and monodehydroascorbate reductase (MDHAR) with NADPH as the reducing power. This may lead to the alteration of redox potential of cells, and consequently membrane destabilization under stress (Noctor and Foyer, 1998; Mittler, 2002). BRs probably maintain the modified cell redox state by regulating activities of SOD, CAT, APOX, glutathione reductase (GR), DHAR, and MDHAR. The redox potential can be re-established by the means of reducing phospholipid peroxidation in cell membranes or by the accumulation of certain osmoprotectant, e.g., proline (**Figure 1**). Fariduddin et al. (2009) reported that the content of proline and antioxidative enzymes increased in response to copper stress, and this effect became even stronger after the application of HBL. Furthermore, BRs can modulate the activity of proteins and other enzymes in the membrane, either influencing protein conformation or protein activity by direct interactions of proteins and sterols (Lindsey et al., 2003). It is known that BRs are recognized by a protein complex that includes the leucine-rich repeat receptor-like kinase encodes by BRI1. BRI1 can receive peptide signals, thus serving the protective role (Wang et al., 2014). At the occurrence of stress, these signals may regulate defense responses. Different BR-regulated genes participate in the response to stress and may encode PC (**Figure 1**), osmolytes, organic acids, metallothioneins, and stress protective proteins such as heat-shock proteins (Gendron and Wang, 2007).

Brassinosteroids may inhibit the degradation of lipids resulting from the overproduction of ROS under metal stress, and stimulate the activity of antioxidative enzymes (**Figure 1**) (Sudo et al., 2008; Soares et al., 2016). Other studies show that increased expression of GR, DHAR, and MDHAR is observed in plants exposed to chromium combined with HBL, which improves their tolerance to oxidative stress caused by this metal. The modified activity of antioxidant enzymes may suggest that HBL treated seedlings were affected by chromium to a lesser extent than those which have not been supplemented with HBL (Sharma et al., 2011). It has also been proved that in *B. juncea* arsenic-related stress causes the activation of antioxidative enzymes and synthesis of BRs, which may be one of plants’ strategies of protection from stress. Synthesis of BRs is either an example of direct impact of metal stress or a response of *B. juncea* plants to the stress, aimed at alleviating its effects (Kanwar et al., 2015).

## Phytochelatins Synthesis

The syntheses of PC are one of the mechanism of metal detoxification in plants. PC are metal-binding cysteine-rich compounds, which facilitate the chelation of metal ions in cytosol or their compartmentalization in vacuoles (**Figure 1**). Their general structure is (γ-Glu-Cys)*_n_*-Gly, where *n* can be as high as 11, but it is usually in the range of 2–5. These metal-binding peptides are derived from glutathione. BRs are known to stimulate the syntheses of PC in cells treated with lead (Bajguz, 2002).

## Biosorption

Biosorption is another mechanism used by plants to remove metals, independent of cell metabolism. Binding metal ions may occur through the mechanism of physical adsorption, ion exchange, chemical sorption, complexation, or chelatation. The metal ions are adsorbed by biosorbents as a result of their interactions with functional (carboxyl, hydroxyl, amino, phosphoryl, etc.) groups present on the surface of the biosorbent’s cell wall. Research on the usefulness of sorption in removing metal ions from water solutions proves that the pH of the solution is particularly important for the process. The optimum pH of metal ions sorption for most of the natural sorbents is within the range of 4–6 (Han et al., 2005; Gupta et al., 2009). Lowering the pH in cell wall spaces stimulates the growth of *C. vulgaris* under the influence of BRs. The impact of BRs on proton excretion is related to early hyperpolarization of transmembrane electrical potential. Proton excretion caused by these hormones is additionally stimulated by the presence of K^+^ in the substrate (Bajguz, 2002).

## Conclusion

Plants under various stresses, both biotic and abiotic ones, generate large amounts of ROS which may oxidize lipids, proteins, and nucleic acids, resulting in disturbances at the cellular level which lead to apoptosis. The toxic influence of metals on plants also depends on its absorption and bioaccumulation, which in turn is associated with the bioavailability, uptake route, storage, degradation, immobilization, and excretion of the metal, as well as avoidance/tolerance mechanisms. Studies concerning the molecular mechanism of BRs’ activity will allow for better and more thorough understanding of anti-stress activities of these phytohormones and facilitate the development of appropriate strategies of protecting plants from metal stress. BR signal transduction may result in activation of NADPH oxidase to ROS production, which probably initiates a cascade of protein phosphorylation through MAPKs to activate transcription factors to target specific genes participating in cellular protection (Xia et al., 2009). Owing to the importance of BRs in the crop improvement, further investigations are needed to identify the key regulatory elements in BR signaling pathway and the underlying mechanism of BR-modulated growth and developmental responses in major crop plants to design optimal strategies to enhance crop yield and improve their performance under stress conditions.

## Perspectives

Care about the environment and the necessity to restore its original properties forces people to seek new, alternative ways of cleaning the environment. A cheap and promising way of solving ecological problems is phytoremediation. Assisted phytoremediation is based on chemical additives to soil, such as chelators, organic acids, etc., which improve the bioavailability of metals for plant uptake. Some recent studies are focused on the use of phytohormones as plant additives for phytoremediation purposes (Barbafieri and Tassi, 2011). Because BRs can modify some agronomic traits of plants, the application of these phytohormones in phytoremediation is a desired subject of study.

## Author Contributions

IR and MT contributed to the writing of the manuscript. AB participated in drawing **Figure 1** and in critically revising the manuscript.

## Conflict of Interest Statement

The authors declare that the research was conducted in the absence of any commercial or financial relationships that could be construed as a potential conflict of interest.
